# *N*-[(6-Bromo-2-meth­oxy-3-quinol­yl)phenyl­meth­yl]-2-morpholino-*N*-(1-phenyl­ethyl)acetamide

**DOI:** 10.1107/S1600536809027020

**Published:** 2009-07-18

**Authors:** Zhi-Qiang Cai, Gang Xiong, Shan-Rong Li, Jian-Bo Liu, Tie-Min Sun

**Affiliations:** a College of Pharmaceutical Engineering, Shenyang Pharmaceutical University, Shenyang 110016, People’s Republic of China; b Laboratory of Coordination Chemistry, Shenyang Institute of Chemical Technology, Shenyang 110142, People’s Republic of China

## Abstract

In the title compound, C_31_H_32_BrN_3_O_3_, the morpholine ring adopts a chair conformation, and the planar quinoline system is twisted with respect to the phenyl rings, with dihedral angles of 17.6 (4) and 75.1 (3)°. Intramolecular C—H⋯O and C—H⋯N hydrogen bonds are present. The crystal packing is stabilized by weak C—H⋯O hydrogen bonding and C—H⋯π interactions.

## Related literature

For the synthesis of other phamaceutically active derivatives through conventional and other synthetic routes, see: Andries *et al.* (2005 ▶); Gaurrand *et al.* (2006 ▶); Mao *et al.* (2007 ▶); Dalla Via *et al.* (2008 ▶). For related structures, see: Petit *et al.* (2007 ▶); Rahmani *et al.* (2009 ▶).
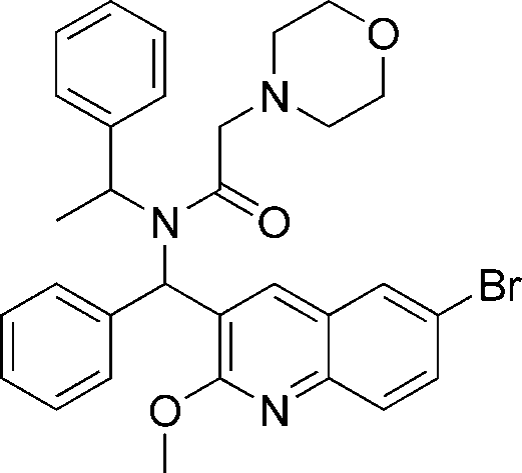


## Experimental

### 

#### Crystal data

C_31_H_32_BrN_3_O_3_
*M* *_r_* = 574.50
Triclinic, 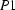

*a* = 7.8696 (14) Å
*b* = 9.4558 (16) Å
*c* = 9.8018 (17) Å
α = 90.544 (2)°
β = 100.562 (3)°
γ = 104.618 (2)°
*V* = 692.6 (2) Å^3^
*Z* = 1
Mo *K*α radiation
μ = 1.52 mm^−1^
*T* = 298 K
0.45 × 0.33 × 0.31 mm

#### Data collection

Bruker SMART APEX CCD diffractometer
Absorption correction: multi-scan (*SADABS*; Bruker, 2001 ▶) *T*_min_ = 0.525, *T*_max_ = 0.625
4628 measured reflections
3796 independent reflections
2917 reflections with *I* > 2σ(*I*)
*R*_int_ = 0.019

#### Refinement

*R*[*F* ^2^ > 2σ(*F* ^2^)] = 0.076
*wR*(*F* ^2^) = 0.236
*S* = 1.02
3796 reflections
346 parameters
4 restraints
H-atom parameters constrained
Δρ_max_ = 0.66 e Å^−3^
Δρ_min_ = −0.47 e Å^−3^
Absolute structure: Flack (1983 ▶), 1149 Friedel pairs
Flack parameter: 0.14 (2)

### 

Data collection: *SMART* (Bruker, 2001 ▶); cell refinement: *SAINT* (Bruker, 2001 ▶); data reduction: *SAINT*; program(s) used to solve structure: *SHELXTL* (Sheldrick, 2008 ▶); program(s) used to refine structure: *SHELXTL*; molecular graphics: *SHELXTL*; software used to prepare material for publication: *SHELXTL*.

## Supplementary Material

Crystal structure: contains datablocks I, global. DOI: 10.1107/S1600536809027020/xu2541sup1.cif

Structure factors: contains datablocks I. DOI: 10.1107/S1600536809027020/xu2541Isup2.hkl

Additional supplementary materials:  crystallographic information; 3D view; checkCIF report

## Figures and Tables

**Table 1:** Hydrogen-bond geometry (Å, °)

*D*—H⋯*A*	*D*—H	H⋯*A*	*D*⋯*A*	*D*—H⋯*A*
C8—H8⋯N3	0.98	2.55	3.105 (7)	116
C25—H25⋯O2	0.93	2.45	3.153 (7)	133
C30—H30⋯O2^i^	0.93	2.59	3.399 (10)	145
C7—H7*B*⋯*Cg*4^ii^	0.96	3.15	3.988 (8)	147
C23—H23⋯*Cg*5^iii^	0.93	2.79	3.662 (8)	157

Symmetry codes: (i) 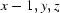; (ii) 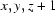; (iii) 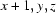. *Cg*4 and *Cg*5 are the centroids of the O3,C1,C2,N3,C3,C4 and C27–C32 rings, respectively.

**Fig. 1.**
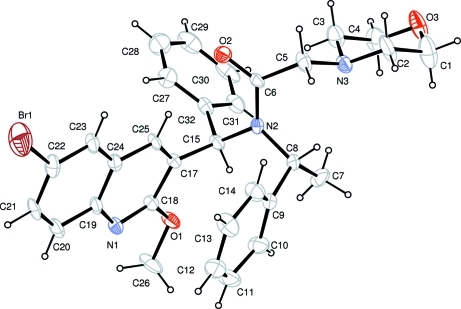
The structure of the title compound with all non-H atom-labeling scheme and ellipsoids drawn at the 30% probability level.

## supplementary crystallographic information

### Comment

The quinolines and their derivatives as a class of extremely important heterocyclic compounds are used in a wide array of synthetic and medcinal applications. Some quinoline derivatives can give effective and good quality drugs treating many cancers (Dalla Via *et al.*, 2008), and also used as antifungals and antituberculostatics drugs (Andries *et al.*, 2005; Mao *et al.*, 2007; Gaurrand *et al.*, 2006). We report herein the crystal structure of the title compound.

In the molecule of the title compound (Fig 1), the morpholine system exists in a chair conformation. The dihedral angles of aromatic rings are different from related structure TMC-207 (Petit *et al.*, 2007; Rahmani *et al.*, 2009), which has been completed Phase II clinical, and will be marked in 2012 as a kind of antituberculostatics drug. The dihedral angle between phenyl ring [C9—C14] and substituted quinolinyl groups is 17.6 (4)°, the dihedral angle between phenyl ring [C27—C32] and substituted quinolinyl groups is 75.1 (3)°, and the dihedral angle between phenyl ring [C9—C14] and phenyl ring[C27—C32] is 68.3 (4)°; while the dihedral angles between phenyl and substituted quinolinyl groups in related structure TMC-207 is 97.4°, and naphthalenyl and substituted quinolinyl groups is nearly coplanar. The distance of the centroids between the phenyl ring [C9—C14] and the ring containing N of the substituted quinolinyl groups is 3.598 Å. The results suggest that the differences between them are caused by steric three-dimensional space. The structural cohesion of the title compound is ensured by weaker contacts. The C—H···O and C—H···N hydrogen bonding is present in the crystal structure (Table 1).

### Experimental

To a solution of *N*-((6-bromo-2-methoxyquinolin-3-yl)phenylmethyl)-2- chloro-*N*-(1-phenylethyl)acetamide (1 mmol) and potassium carbonate (5 mmol) in acetonitrile (50 ml), morpholine (1 mmol) was added. The mixture was stirred for 6 h below 353 K. The reaction mixture was left for 2 h and then diluted with water (50 ml). The resulting precipitate was collected by extraction, distillation and purified on silica gel column (30% ethyl acetate in hexane) to give white powder (91.2% yield). Crystals suitable for X-ray analysis were obtained from acetonitrile solution by slow evaporation.

### Refinement

All H atoms were geometrically positioned (C—H 0.93–0.98 Å) and treated as riding, with *U*_iso_(H) = 1.5U_eq_(C) for methyl H atoms and 1.2U_eq_(C) for the others.

### Crystal data

**Table:**

C_31_H_32_BrN_3_O_3_	*Z* = 1
*M**_r_* = 574.50	*F*(000) = 298
Triclinic, *P*1	*D*_x_ = 1.377 Mg m^−^^3^
Hall symbol: P 1	Mo *K*α radiation, λ = 0.71073 Å
*a* = 7.8696 (14) Å	Cell parameters from 3537 reflections
*b* = 9.4558 (16) Å	θ = 2.1–25.9°
*c* = 9.8018 (17) Å	µ = 1.52 mm^−^^1^
α = 90.544 (2)°	*T* = 298 K
β = 100.562 (3)°	Prism, colorless
γ = 104.618 (2)°	0.45 × 0.33 × 0.31 mm
*V* = 692.6 (2) Å^3^	

### Data collection

**Table:**

Bruker SMART APEX CCD diffractometer	3796 independent reflections
Radiation source: fine-focus sealed tube	2917 reflections with *I* > 2σ(*I*)
graphite	*R*_int_ = 0.019
φ and ω scans	θ_max_ = 25.9°, θ_min_ = 2.1°
Absorption correction: multi-scan (*SADABS*; Bruker, 2001)	*h* = −9→8
*T*_min_ = 0.525, *T*_max_ = 0.625	*k* = −11→11
4628 measured reflections	*l* = −10→12

### Refinement

**Table:**

Refinement on *F*^2^	Secondary atom site location: difference Fourier map
Least-squares matrix: full	Hydrogen site location: inferred from neighbouring sites
*R*[*F*^2^ > 2σ(*F*^2^)] = 0.076	H-atom parameters constrained
*wR*(*F*^2^) = 0.236	*w* = 1/[σ^2^(*F*_o_^2^) + (0.1743*P*)^2^] where *P* = (*F*_o_^2^ + 2*F*_c_^2^)/3
*S* = 1.02	(Δ/σ)_max_ < 0.001
3796 reflections	Δρ_max_ = 0.66 e Å^−^^3^
346 parameters	Δρ_min_ = −0.46 e Å^−^^3^
4 restraints	Absolute structure: Flack (1983), 1149 Friedel pairs
Primary atom site location: structure-invariant direct methods	Flack parameter: 0.14 (2)

### Special details

**Table:**

Geometry. All e.s.d.'s (except the e.s.d. in the dihedral angle between two l.s. planes) are estimated using the full covariance matrix. The cell e.s.d.'s are taken into account individually in the estimation of e.s.d.'s in distances, angles and torsion angles; correlations between e.s.d.'s in cell parameters are only used when they are defined by crystal symmetry. An approximate (isotropic) treatment of cell e.s.d.'s is used for estimating e.s.d.'s involving l.s. planes.
Refinement. Refinement of *F*^2^ against ALL reflections. The weighted *R*-factor *wR* and goodness of fit *S* are based on *F*^2^, conventional *R*-factors *R* are based on *F*, with *F* set to zero for negative *F*^2^. The threshold expression of *F*^2^ > σ(*F*^2^) is used only for calculating *R*-factors(gt) *etc*. and is not relevant to the choice of reflections for refinement. *R*-factors based on *F*^2^ are statistically about twice as large as those based on *F*, and *R*- factors based on ALL data will be even larger.

### Fractional atomic coordinates and isotropic or equivalent isotropic displacement parameters (Å^2^)

**Table:**

	*x*	*y*	*z*	*U*_iso_*/*U*_eq_	
Br1	1.24839 (17)	0.39015 (15)	−0.05433 (14)	0.1036 (5)	
C4	0.3479 (12)	−0.0267 (12)	0.8340 (10)	0.072 (2)	
H4A	0.3364	0.0712	0.8523	0.087*	
H4B	0.2288	−0.0899	0.8016	0.087*	
C3	0.4565 (11)	−0.0236 (10)	0.7234 (9)	0.0593 (19)	
H3A	0.4610	−0.1223	0.6999	0.071*	
H3B	0.4007	0.0147	0.6404	0.071*	
C1	0.5983 (15)	0.0096 (16)	1.0047 (11)	0.093 (3)	
H1A	0.6513	−0.0286	1.0889	0.111*	
H1B	0.5928	0.1083	1.0274	0.111*	
C2	0.7147 (14)	0.0142 (12)	0.8971 (10)	0.078 (3)	
H2A	0.8328	0.0777	0.9322	0.093*	
H2B	0.7273	−0.0833	0.8788	0.093*	
C5	0.7473 (10)	0.0755 (8)	0.6650 (7)	0.0459 (16)	
H5A	0.7580	−0.0218	0.6433	0.055*	
H5B	0.8664	0.1365	0.7020	0.055*	
C6	0.6689 (8)	0.1390 (6)	0.5298 (6)	0.0317 (12)	
C7	0.5793 (12)	0.4161 (8)	0.7307 (9)	0.060 (2)	
H7A	0.5232	0.4804	0.6745	0.090*	
H7B	0.6332	0.4632	0.8210	0.090*	
H7C	0.4911	0.3276	0.7400	0.090*	
C8	0.7214 (9)	0.3799 (6)	0.6625 (6)	0.0373 (14)	
H8	0.7906	0.3306	0.7307	0.045*	
C9	0.8530 (10)	0.5077 (7)	0.6180 (6)	0.0398 (14)	
C14	1.0104 (10)	0.4857 (8)	0.5883 (9)	0.0508 (16)	
H14	1.0386	0.3971	0.6065	0.061*	
C13	1.1252 (11)	0.5939 (9)	0.5320 (10)	0.059 (2)	
H13	1.2287	0.5758	0.5112	0.070*	
C12	1.0920 (12)	0.7264 (8)	0.5058 (10)	0.063 (2)	
H12	1.1741	0.7998	0.4721	0.076*	
C11	0.9277 (13)	0.7502 (8)	0.5314 (11)	0.066 (2)	
H11	0.8944	0.8352	0.5058	0.079*	
C10	0.8187 (11)	0.6417 (7)	0.5959 (9)	0.0532 (18)	
H10	0.7201	0.6609	0.6246	0.064*	
C15	0.5369 (8)	0.3306 (6)	0.4201 (6)	0.0350 (13)	
H15	0.4995	0.4106	0.4600	0.042*	
C17	0.6440 (8)	0.4009 (6)	0.3131 (6)	0.0332 (13)	
C18	0.6119 (8)	0.5339 (6)	0.2557 (6)	0.0328 (12)	
C19	0.8276 (8)	0.5586 (6)	0.1238 (6)	0.0353 (13)	
C20	0.9212 (10)	0.6379 (8)	0.0287 (7)	0.0461 (16)	
H20	0.8967	0.7252	−0.0003	0.055*	
C21	1.0491 (11)	0.5886 (8)	−0.0229 (8)	0.0540 (18)	
H21	1.1107	0.6423	−0.0859	0.065*	
C22	1.0847 (9)	0.4566 (8)	0.0209 (7)	0.0498 (17)	
C23	0.9987 (10)	0.3773 (7)	0.1165 (7)	0.0419 (14)	
H23	1.0267	0.2912	0.1457	0.050*	
C24	0.8690 (8)	0.4261 (6)	0.1699 (6)	0.0357 (13)	
C25	0.7735 (8)	0.3516 (6)	0.2667 (6)	0.0323 (12)	
H25	0.7992	0.2662	0.3004	0.039*	
C26	0.4505 (13)	0.7175 (8)	0.2544 (10)	0.067 (2)	
H26A	0.5093	0.7945	0.3245	0.101*	
H26B	0.3242	0.7092	0.2360	0.101*	
H26C	0.4966	0.7396	0.1707	0.101*	
C31	0.2225 (10)	0.1953 (8)	0.4322 (9)	0.0499 (17)	
H31	0.2364	0.2511	0.5141	0.060*	
C30	0.0646 (10)	0.0914 (9)	0.3835 (11)	0.062 (2)	
H30	−0.0274	0.0771	0.4335	0.074*	
C29	0.0405 (10)	0.0078 (10)	0.2614 (12)	0.065 (2)	
H29	−0.0676	−0.0608	0.2286	0.078*	
C28	0.1781 (13)	0.0275 (10)	0.1892 (11)	0.072 (3)	
H28	0.1658	−0.0303	0.1090	0.086*	
C27	0.3378 (8)	0.1360 (7)	0.2379 (8)	0.0468 (16)	
H27	0.4287	0.1530	0.1867	0.056*	
C32	0.3609 (9)	0.2165 (7)	0.3588 (7)	0.0383 (14)	
N1	0.7011 (8)	0.6132 (5)	0.1700 (6)	0.0387 (12)	
N2	0.6404 (7)	0.2747 (5)	0.5409 (5)	0.0327 (10)	
N3	0.6363 (7)	0.0673 (5)	0.7712 (6)	0.0411 (12)	
O1	0.4833 (7)	0.5790 (4)	0.3030 (5)	0.0468 (12)	
O2	0.6376 (6)	0.0666 (4)	0.4219 (5)	0.0418 (10)	
O3	0.4266 (10)	−0.0772 (9)	0.9568 (7)	0.088 (2)	

### Atomic displacement parameters (Å^2^)

**Table:**

	*U*^11^	*U*^22^	*U*^33^	*U*^12^	*U*^13^	*U*^23^
Br1	0.1037 (8)	0.1453 (11)	0.0862 (8)	0.0497 (7)	0.0549 (6)	0.0297 (7)
C4	0.056 (5)	0.088 (6)	0.069 (6)	−0.001 (4)	0.029 (4)	0.021 (5)
C3	0.058 (5)	0.071 (5)	0.048 (4)	0.009 (4)	0.017 (3)	0.017 (4)
C1	0.074 (7)	0.142 (9)	0.057 (6)	0.010 (7)	0.025 (5)	0.039 (6)
C2	0.090 (7)	0.089 (6)	0.050 (5)	0.011 (5)	0.016 (5)	0.042 (5)
C5	0.052 (4)	0.058 (4)	0.041 (4)	0.028 (3)	0.024 (3)	0.021 (3)
C6	0.036 (3)	0.029 (3)	0.035 (3)	0.010 (2)	0.018 (2)	0.012 (2)
C7	0.077 (5)	0.050 (4)	0.053 (5)	−0.005 (4)	0.043 (4)	−0.015 (3)
C8	0.049 (4)	0.035 (3)	0.027 (3)	0.005 (3)	0.014 (3)	0.004 (2)
C9	0.055 (4)	0.037 (3)	0.026 (3)	0.008 (3)	0.009 (3)	0.001 (2)
C14	0.048 (4)	0.049 (4)	0.058 (5)	0.011 (3)	0.020 (3)	0.012 (3)
C13	0.039 (4)	0.071 (5)	0.073 (6)	0.015 (3)	0.029 (4)	0.024 (4)
C12	0.071 (5)	0.049 (4)	0.064 (5)	−0.002 (4)	0.021 (4)	0.004 (4)
C11	0.080 (6)	0.039 (3)	0.084 (6)	0.006 (4)	0.042 (5)	0.015 (4)
C10	0.061 (4)	0.041 (3)	0.067 (5)	0.015 (3)	0.034 (4)	0.001 (3)
C15	0.043 (3)	0.033 (3)	0.037 (3)	0.017 (2)	0.020 (3)	0.014 (2)
C17	0.042 (3)	0.028 (3)	0.029 (3)	0.005 (2)	0.009 (2)	0.005 (2)
C18	0.032 (3)	0.032 (3)	0.034 (3)	0.007 (2)	0.010 (2)	0.007 (2)
C19	0.039 (3)	0.039 (3)	0.024 (3)	0.001 (2)	0.008 (2)	0.008 (2)
C20	0.052 (4)	0.048 (3)	0.038 (4)	0.005 (3)	0.018 (3)	0.019 (3)
C21	0.071 (5)	0.058 (4)	0.038 (4)	0.009 (4)	0.033 (3)	0.026 (3)
C22	0.044 (4)	0.071 (5)	0.035 (4)	0.010 (3)	0.018 (3)	0.008 (3)
C23	0.048 (4)	0.051 (3)	0.032 (3)	0.016 (3)	0.019 (3)	0.014 (3)
C24	0.036 (3)	0.040 (3)	0.028 (3)	0.005 (2)	0.005 (2)	0.005 (2)
C25	0.037 (3)	0.035 (3)	0.032 (3)	0.011 (2)	0.020 (2)	0.017 (2)
C26	0.087 (6)	0.051 (4)	0.093 (7)	0.046 (4)	0.051 (5)	0.045 (4)
C31	0.056 (4)	0.052 (4)	0.058 (4)	0.029 (3)	0.031 (3)	0.023 (3)
C30	0.034 (4)	0.061 (4)	0.102 (7)	0.017 (3)	0.033 (4)	0.048 (5)
C29	0.036 (4)	0.064 (5)	0.090 (7)	0.003 (3)	0.012 (4)	0.031 (5)
C28	0.067 (6)	0.066 (5)	0.065 (6)	−0.002 (4)	−0.003 (5)	0.000 (4)
C27	0.026 (3)	0.058 (4)	0.055 (4)	0.000 (3)	0.018 (3)	0.003 (3)
C32	0.036 (3)	0.044 (3)	0.040 (4)	0.014 (3)	0.015 (3)	0.017 (3)
N1	0.045 (3)	0.037 (2)	0.034 (3)	0.005 (2)	0.015 (2)	0.009 (2)
N2	0.040 (3)	0.035 (2)	0.025 (2)	0.009 (2)	0.013 (2)	0.0047 (19)
N3	0.047 (3)	0.041 (3)	0.038 (3)	0.011 (2)	0.014 (2)	0.012 (2)
O1	0.062 (3)	0.036 (2)	0.055 (3)	0.023 (2)	0.030 (2)	0.021 (2)
O2	0.049 (3)	0.037 (2)	0.044 (3)	0.0130 (19)	0.018 (2)	0.0039 (19)
O3	0.090 (5)	0.117 (5)	0.062 (4)	0.017 (4)	0.036 (4)	0.051 (4)

### Geometric parameters (Å, °)

**Table:**

Br1—C22	1.829 (8)	C15—N2	1.490 (8)
C4—O3	1.396 (12)	C15—C17	1.519 (9)
C4—C3	1.495 (13)	C15—C32	1.540 (9)
C4—H4A	0.9700	C15—H15	0.9800
C4—H4B	0.9700	C17—C25	1.367 (9)
C3—N3	1.448 (10)	C17—C18	1.444 (8)
C3—H3A	0.9700	C18—N1	1.313 (8)
C3—H3B	0.9700	C18—N1	1.313 (8)
C1—O3	1.386 (13)	C18—O1	1.344 (8)
C1—C2	1.513 (16)	C19—N1	1.372 (9)
C1—H1A	0.9700	C19—N1	1.372 (9)
C1—H1B	0.9700	C19—C20	1.400 (9)
C2—N3	1.430 (10)	C19—C24	1.429 (8)
C2—H2A	0.9700	C20—C21	1.380 (11)
C2—H2B	0.9700	C20—H20	0.9300
C5—N3	1.468 (9)	C21—C22	1.402 (11)
C5—C6	1.549 (8)	C21—H21	0.9300
C5—H5A	0.9700	C22—C23	1.374 (10)
C5—H5B	0.9700	C23—C24	1.400 (10)
C6—O2	1.206 (8)	C23—H23	0.9300
C6—O2	1.206 (8)	C24—C25	1.403 (9)
C6—N2	1.364 (7)	C25—H25	0.9300
C7—C8	1.509 (10)	C26—O1	1.465 (7)
C7—H7A	0.9600	C26—H26A	0.9600
C7—H7B	0.9600	C26—H26B	0.9600
C7—H7C	0.9600	C26—H26C	0.9600
C8—N2	1.482 (8)	C31—C30	1.377 (12)
C8—C9	1.506 (9)	C31—C32	1.387 (10)
C8—H8	0.9800	C31—H31	0.9300
C9—C10	1.372 (9)	C30—C29	1.384 (15)
C9—C14	1.387 (10)	C30—H30	0.9300
C14—C13	1.376 (12)	C29—C28	1.375 (14)
C14—H14	0.9300	C29—H29	0.9300
C13—C12	1.361 (12)	C28—C27	1.407 (11)
C13—H13	0.9300	C28—H28	0.9300
C12—C11	1.431 (13)	C27—C32	1.360 (11)
C12—H12	0.9300	C27—H27	0.9300
C11—C10	1.398 (12)	N1—N1	0.000
C11—H11	0.9300	O2—O2	0.000
C10—H10	0.9300		
			
O3—C4—C3	111.4 (8)	N2—C15—H15	104.8
O3—C4—H4A	109.3	C17—C15—H15	104.8
C3—C4—H4A	109.3	C32—C15—H15	104.8
O3—C4—H4B	109.3	C25—C17—C18	115.7 (5)
C3—C4—H4B	109.3	C25—C17—C15	125.9 (5)
H4A—C4—H4B	108.0	C18—C17—C15	118.4 (5)
N3—C3—C4	110.2 (7)	N1—C18—O1	119.9 (5)
N3—C3—H3A	109.6	N1—C18—O1	119.9 (5)
C4—C3—H3A	109.6	N1—C18—C17	125.8 (6)
N3—C3—H3B	109.6	N1—C18—C17	125.8 (6)
C4—C3—H3B	109.6	O1—C18—C17	114.1 (5)
H3A—C3—H3B	108.1	N1—C19—C20	118.1 (6)
O3—C1—C2	111.4 (10)	N1—C19—C20	118.1 (6)
O3—C1—H1A	109.4	N1—C19—C24	122.9 (6)
C2—C1—H1A	109.4	N1—C19—C24	122.9 (6)
O3—C1—H1B	109.4	C20—C19—C24	118.9 (6)
C2—C1—H1B	109.4	C21—C20—C19	121.2 (6)
H1A—C1—H1B	108.0	C21—C20—H20	119.4
N3—C2—C1	110.1 (8)	C19—C20—H20	119.4
N3—C2—H2A	109.6	C20—C21—C22	119.0 (6)
C1—C2—H2A	109.6	C20—C21—H21	120.5
N3—C2—H2B	109.6	C22—C21—H21	120.5
C1—C2—H2B	109.6	C23—C22—C21	121.6 (7)
H2A—C2—H2B	108.2	C23—C22—Br1	120.3 (6)
N3—C5—C6	112.5 (5)	C21—C22—Br1	118.1 (5)
N3—C5—H5A	109.1	C22—C23—C24	119.9 (6)
C6—C5—H5A	109.1	C22—C23—H23	120.0
N3—C5—H5B	109.1	C24—C23—H23	120.0
C6—C5—H5B	109.1	C23—C24—C25	123.8 (5)
H5A—C5—H5B	107.8	C23—C24—C19	119.3 (6)
O2—C6—N2	124.1 (5)	C25—C24—C19	116.9 (6)
O2—C6—N2	124.1 (5)	C17—C25—C24	121.9 (5)
O2—C6—C5	118.5 (5)	C17—C25—H25	119.0
O2—C6—C5	118.5 (5)	C24—C25—H25	119.0
N2—C6—C5	117.4 (5)	O1—C26—H26A	109.5
C8—C7—H7A	109.5	O1—C26—H26B	109.5
C8—C7—H7B	109.5	H26A—C26—H26B	109.5
H7A—C7—H7B	109.5	O1—C26—H26C	109.5
C8—C7—H7C	109.5	H26A—C26—H26C	109.5
H7A—C7—H7C	109.5	H26B—C26—H26C	109.5
H7B—C7—H7C	109.5	C30—C31—C32	119.8 (8)
N2—C8—C9	108.4 (5)	C30—C31—H31	120.1
N2—C8—C7	111.1 (5)	C32—C31—H31	120.1
C9—C8—C7	116.3 (5)	C31—C30—C29	121.1 (8)
N2—C8—H8	106.9	C31—C30—H30	119.4
C9—C8—H8	106.9	C29—C30—H30	119.4
C7—C8—H8	106.9	C28—C29—C30	119.2 (8)
C10—C9—C14	118.5 (7)	C28—C29—H29	120.4
C10—C9—C8	123.0 (6)	C30—C29—H29	120.4
C14—C9—C8	118.3 (6)	C29—C28—C27	119.4 (9)
C13—C14—C9	120.5 (7)	C29—C28—H28	120.3
C13—C14—H14	119.7	C27—C28—H28	120.3
C9—C14—H14	119.7	C32—C27—C28	120.9 (8)
C12—C13—C14	122.1 (8)	C32—C27—H27	119.5
C12—C13—H13	119.0	C28—C27—H27	119.5
C14—C13—H13	119.0	C27—C32—C31	119.5 (6)
C13—C12—C11	118.4 (8)	C27—C32—C15	122.7 (6)
C13—C12—H12	120.8	C31—C32—C15	117.7 (6)
C11—C12—H12	120.8	C18—N1—C19	116.6 (5)
C10—C11—C12	118.1 (7)	C6—N2—C8	124.1 (5)
C10—C11—H11	121.0	C6—N2—C15	119.7 (5)
C12—C11—H11	121.0	C8—N2—C15	115.9 (4)
C9—C10—C11	121.9 (7)	C2—N3—C3	109.3 (6)
C9—C10—H10	119.0	C2—N3—C5	111.6 (6)
C11—C10—H10	119.0	C3—N3—C5	112.2 (6)
N2—C15—C17	115.4 (5)	C18—O1—C26	116.8 (5)
N2—C15—C32	111.3 (5)	C1—O3—C4	110.7 (7)
C17—C15—C32	114.6 (5)		
			
O3—C4—C3—N3	−57.5 (10)	C31—C30—C29—C28	1.2 (11)
O3—C1—C2—N3	57.8 (12)	C30—C29—C28—C27	−2.5 (12)
N3—C5—C6—O2	−123.0 (6)	C29—C28—C27—C32	3.3 (12)
N3—C5—C6—O2	−123.0 (6)	C28—C27—C32—C31	−2.6 (10)
N3—C5—C6—N2	57.3 (8)	C28—C27—C32—C15	176.7 (7)
N2—C8—C9—C10	104.9 (7)	C30—C31—C32—C27	1.2 (9)
C7—C8—C9—C10	−21.1 (9)	C30—C31—C32—C15	−178.1 (6)
N2—C8—C9—C14	−70.2 (7)	N2—C15—C32—C27	−104.6 (7)
C7—C8—C9—C14	163.8 (7)	C17—C15—C32—C27	28.5 (8)
C10—C9—C14—C13	−2.9 (11)	N2—C15—C32—C31	74.7 (7)
C8—C9—C14—C13	172.5 (8)	C17—C15—C32—C31	−152.2 (5)
C9—C14—C13—C12	1.3 (13)	O1—C18—N1—N1	0.0 (5)
C14—C13—C12—C11	−3.3 (14)	C17—C18—N1—N1	0.0 (6)
C13—C12—C11—C10	6.8 (13)	N1—C18—N1—C19	0(100)
C14—C9—C10—C11	6.7 (12)	O1—C18—N1—C19	−178.6 (5)
C8—C9—C10—C11	−168.4 (7)	C17—C18—N1—C19	4.8 (9)
C12—C11—C10—C9	−8.7 (13)	C20—C19—N1—N1	0.0 (6)
N2—C15—C17—C25	40.5 (8)	C24—C19—N1—N1	0.0 (5)
C32—C15—C17—C25	−90.7 (7)	N1—C19—N1—C18	0(100)
N2—C15—C17—C18	−137.9 (5)	C20—C19—N1—C18	177.9 (6)
C32—C15—C17—C18	90.9 (6)	C24—C19—N1—C18	−2.9 (8)
C25—C17—C18—N1	−3.5 (9)	O2—C6—N2—C8	−162.8 (6)
C15—C17—C18—N1	175.0 (6)	O2—C6—N2—C8	−162.8 (6)
C25—C17—C18—N1	−3.5 (9)	C5—C6—N2—C8	16.9 (8)
C15—C17—C18—N1	175.0 (6)	O2—C6—N2—C15	10.9 (9)
C25—C17—C18—O1	179.7 (5)	O2—C6—N2—C15	10.9 (9)
C15—C17—C18—O1	−1.7 (7)	C5—C6—N2—C15	−169.4 (5)
N1—C19—C20—C21	−179.4 (6)	C9—C8—N2—C6	112.1 (6)
N1—C19—C20—C21	−179.4 (6)	C7—C8—N2—C6	−119.0 (7)
C24—C19—C20—C21	1.3 (10)	C9—C8—N2—C15	−61.8 (7)
C19—C20—C21—C22	0.2 (11)	C7—C8—N2—C15	67.2 (7)
C20—C21—C22—C23	−1.7 (11)	C17—C15—N2—C6	−83.9 (6)
C20—C21—C22—Br1	177.6 (6)	C32—C15—N2—C6	48.9 (7)
C21—C22—C23—C24	1.6 (11)	C17—C15—N2—C8	90.3 (6)
Br1—C22—C23—C24	−177.7 (5)	C32—C15—N2—C8	−137.0 (5)
C22—C23—C24—C25	179.4 (6)	C1—C2—N3—C3	−56.3 (11)
C22—C23—C24—C19	0.0 (9)	C1—C2—N3—C5	179.0 (8)
N1—C19—C24—C23	179.4 (6)	C4—C3—N3—C2	56.6 (10)
N1—C19—C24—C23	179.4 (6)	C4—C3—N3—C5	−179.1 (7)
C20—C19—C24—C23	−1.4 (8)	C6—C5—N3—C2	−177.6 (7)
N1—C19—C24—C25	−0.1 (8)	C6—C5—N3—C3	59.4 (7)
N1—C19—C24—C25	−0.1 (8)	N1—C18—O1—C26	0.0 (9)
C20—C19—C24—C25	179.1 (6)	N1—C18—O1—C26	0.0 (9)
C18—C17—C25—C24	0.1 (8)	C17—C18—O1—C26	176.9 (6)
C15—C17—C25—C24	−178.3 (6)	N2—C6—O2—O2	0.0 (5)
C23—C24—C25—C17	−178.0 (6)	C5—C6—O2—O2	0.0 (5)
C19—C24—C25—C17	1.4 (8)	C2—C1—O3—C4	−57.8 (13)
C32—C31—C30—C29	−0.5 (10)	C3—C4—O3—C1	58.1 (12)

### Hydrogen-bond geometry (Å, °)

**Table:**

*D*—H···*A*	*D*—H	H···*A*	*D*···*A*	*D*—H···*A*
C8—H8···N3	0.98	2.55	3.105 (7)	116
C25—H25···O2	0.93	2.45	3.153 (7)	133
C15—H15···O1	0.98	2.24	2.722 (8)	109
C30—H30···O2^i^	0.93	2.59	3.399 (10)	145
C7—H7B···Cg4^ii^	0.96	3.15	3.988 (8)	147
C23—H23···Cg5^iii^	0.93	2.79	3.662 (8)	157

Symmetry codes:  (i) *x*−1, *y*, *z*; (ii) *x*, *y*, *z*+1; (iii) *x*+1, *y*, *z*.
